# Recombinant Expression of *Trametes versicolor* Aflatoxin B_1_-Degrading Enzyme (TV-AFB_1_D) in Engineering *Pichia pastoris* GS115 and Application in AFB_1_ Degradation in AFB_1_-Contaminated Peanuts

**DOI:** 10.3390/toxins13050349

**Published:** 2021-05-13

**Authors:** Peizhou Yang, Wei Xiao, Shuhua Lu, Suwei Jiang, Zhi Zheng, Danfeng Zhang, Min Zhang, Shaotong Jiang, Shuying Jiang

**Affiliations:** 1 Anhui Key Laboratory of Intensive Processing of Agricultural Products, College of Food and Biological Engineering, Hefei University of Technology, 420 Feicui Road, Shushan District, Hefei 230601, China; xiaowei2018321@163.com (W.X.); lsh2641715623@163.com (S.L.); zhengzhi@hfut.edu.cn (Z.Z.); zhangdanfeng202104@163.com (D.Z.); zming19861028@126.com (M.Z.); jstxsgj@163.com (S.J.); jiangshuying9805@163.com (S.J.); 2 School of Biological, Food and Environment Engineering, Hefei University, 158 Jinxiu Avenue, Hefei 230601, China; jsuwei0409@163.com

**Keywords:** aflatoxin B_1_, *Pichia pastoris* GS115, peanuts, *Trametes versicolor*, degradation enzyme

## Abstract

Aflatoxins seriously threaten the health of humans and animals due to their potential carcinogenic properties. Enzymatic degradation approach is an effective and environmentally friendly alternative that involves changing the structure of aflatoxins. In this study, *Trametes versicolor* aflatoxin B_1_-degrading enzyme gene (TV-AFB_1_D) was integrated into the genome of *Pichia pastoris* GS115 by homologous recombination approach. The recombinant TV-AFB_1_D was expressed in engineering *P. pastoris* with a size of approximately 77 kDa under the induction of methanol. The maximum activity of TV-AFB_1_D reached 17.5 U/mL after the induction of 0.8% ethanol (*v*/*v*) for 84 h at 28 °C. The AFB_1_ proportion of 75.9% was degraded using AFB_1_ standard sample after catalysis for 12 h. In addition, the AFB_1_ proportion was 48.5% using AFB_1_-contaminated peanuts after the catalysis for 18 h at 34 °C. The recombinant TV-AFB_1_D would have good practical application value in AFB_1_ degradation in food crops. This study provides an alternative degrading enzyme for the degradation of AFB_1_ in aflatoxin-contaminated grain and feed via enzymatic degradation approach.

## 1. Introduction

Aflatoxins are pathogenic fungal toxins produced by *Aspergillus flavus* and *A. parasiticus* [1]. Aflatoxins and their producing bacteria are usually present in food products, feedstuffs, and agricultural raw materials [2]. Aflatoxins are a real threat to human and animal health due to their carcinogenicity and teratogenicity [3]. Among more than 20 kinds of aflatoxin derivatives, AFB_1_ possesses the highest toxicity of liver cancer and immune system damage. AFB_1_ causes carcinogenesis in the presence of the double bond of AFB_1_ furan ring by inhibiting RNA synthesis [4]. The lactone ring of AFB_1_ is the main toxicity site. The cleavage of lactone ring reduces the biological activity of AFB_1_ with the formation of non-fluorescent compound [5]. In addition, a highly reactive AFB_1_-8,9-epoxide capable of reacting with DNA is produced under the activation of cytochrome P450 system [6,7].

Aflatoxins mainly pollute cereals, oils, and their products [8]. As an important agricultural raw material, peanut is directly in touch with *A. flavus* and *A. parasiticus* in the soil [9]. Peanut is highly susceptible to the contamination of aflatoxins. Aflatoxin-contaminated peanuts pollute the environment and cause the serious economic damage [10]. Despite remarkable improvements in the technologies of harvesting and storage processing, the contamination of aflatoxins is still a problem to be solved in the peanut industry [11]. Various physical, chemical, and biological methods have been developed to solve the problem of aflatoxin pollution [12]. The physical approach mainly includes heating treatment [13], UV radiation [14], high temperature [15], and adsorption [16]. The main disadvantages are long treatment time and low degradation rate [17,18]. The chemical methods include oxidant treatment and acid–base method by destroying the structure of aflatoxins [19,20]. Further, both the physical and chemical methods could reduce the quality of nutrition by changing the characteristics of raw materials [21].

Currently, the enzymatic degradation approach has been developed for its high specificity and efficiency of catalysis [22]. Laccase [23], peroxidase [24], and reductase [25] have been proved to be capable of degrading aflatoxins. The edible vegetable oils are vulnerable to aflatoxin pollution in the production process due to the raw materials and processing properties. Aflatoxin B_1_ is one of the most common toxic substances in edible vegetable oil. Recently, the quality and safety of edible vegetable oils has attracted great attention of China State Council and the State Food and Drug Administration. The government is concentrating R&D efforts to solve the problem of aflatoxin pollution.

*Trametes versicolor* is a common polypore mushroom found throughout the world and is also a well-known traditional medicinal mushroom, one that is found growing on tree trunks. We cloned AFB_1_-degrading enzyme gene from *Trametes versicolor* (TV-AFB_1_D) according to Genbank accession number txid717944 in NCBI. *TV-AFB_1_D* was expressed in engineering *E. coli* BL21(DE3) with TV-AFB_1_D activity of 9.3 U/mL (not reported). To further investigate the effectiveness of expression, we integrated TV-AFB_1_D into the genome of *P. pastoris* GS115 in this study. In addition, the application of TV-AFB_1_D for degradation was also investigated in AFB_1_-contaminated peanuts. This study provides a new AFB_1_-degrading enzyme for application in enzymatic degradation in the food and feed industry.

## 2. Results

### 2.1. TV-AFB_1_D Cloning and Evolutionary Tree Analysis

**Figure 1:**
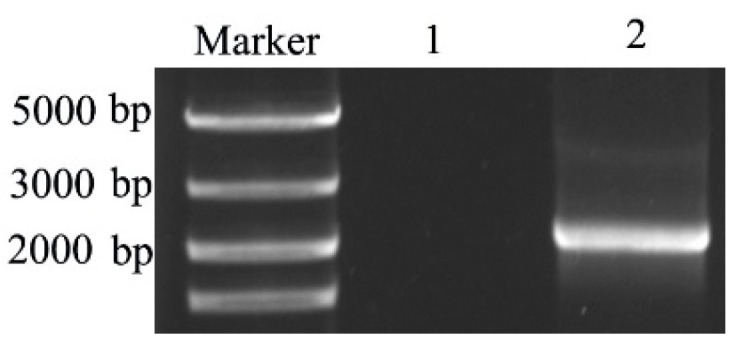
RT-PCR amplification of TV-AFB_1_D. Note: lane 1: control; lane 2: TV-AFB_1_D.

**Figure 2:**
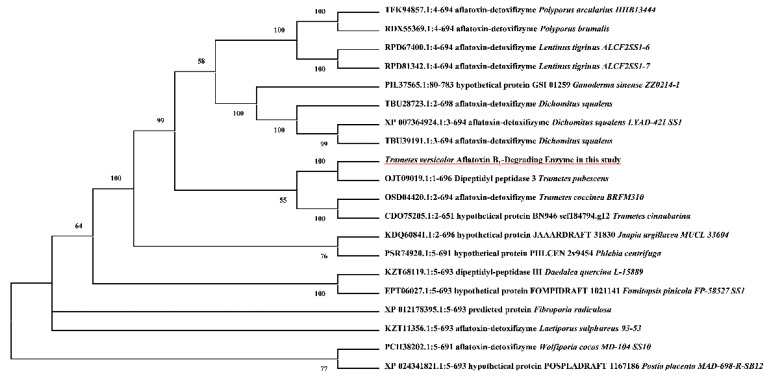
Evolutionary tree analysis of TV-AFB_1_D with other AFB_1_-degrading enzymes.

Aflatoxin B_1_-degrading enzyme TV-AFB_1_D from *T. versicolor* was cloned by using the designed primers with RT-PCR technique (Figure 1). The size of *TV-AFB_1_D* was 2100 bp according to electrophoresis analysis and sequencing confirmation. The evolutionary tree analysis of TV-AFB_1_D with AFB_1_ degradation enzymes from other species is shown in Figure 2. TV-AFB_1_D had closer genetic distance with *Trametes coccinea* than other aflatoxin-degrading enzymes from *Dichomitus squalens*, *Polyporus arcularius*, *Lentinus tigrinus*, and *Laetiporus sulpbureus*. All the aflatoxin-degrading enzymes from *Trametes* genus belonging to family Polyporaceae speculated that aflatoxin-degrading enzymes would have common traits according to the result of evolutionary tree analysis.

### 2.2. Expression Vector Construction of TV-AFB_1_D

**Figure 3:**
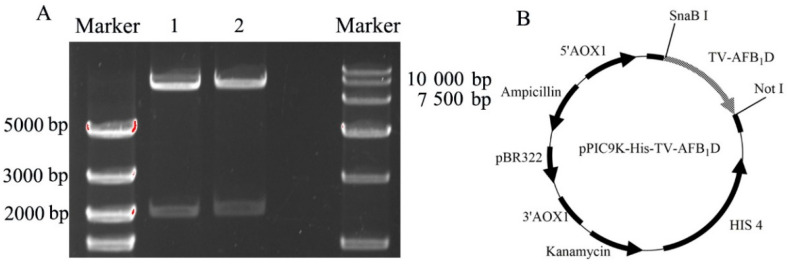
Vector pPIC9K-His-TV-AFB_1_D construction. (**A**) Double-restriction digestion of vector pPIC9K-His-TV-AFB_1_D by *SnaB* I and *Not* I. Note: lanes 1, 2: pPIC9k-TV-AFB_1_D. (**B**) Schematic diagram of constructed pPIC9k-TV-AFB_1_D.

TV-AFB_1_D was integrated into pPIC9K-His vector by double digestion techniques. The constructed recombinant plasmid was identified by the double digestion of *Sna B* I and *Not* I. Two bands after digestion possessed the size of approximately 9300 and 2100 bp (Figure 3A). The confirmed recombinant vector was named pPIC9K-His-AFB_1_D after confirmation by gene sequencing (Figure 3B).

### 2.3. Transformation of TV-AFB_1_D into P. pastoris GS115

**Figure 4:**
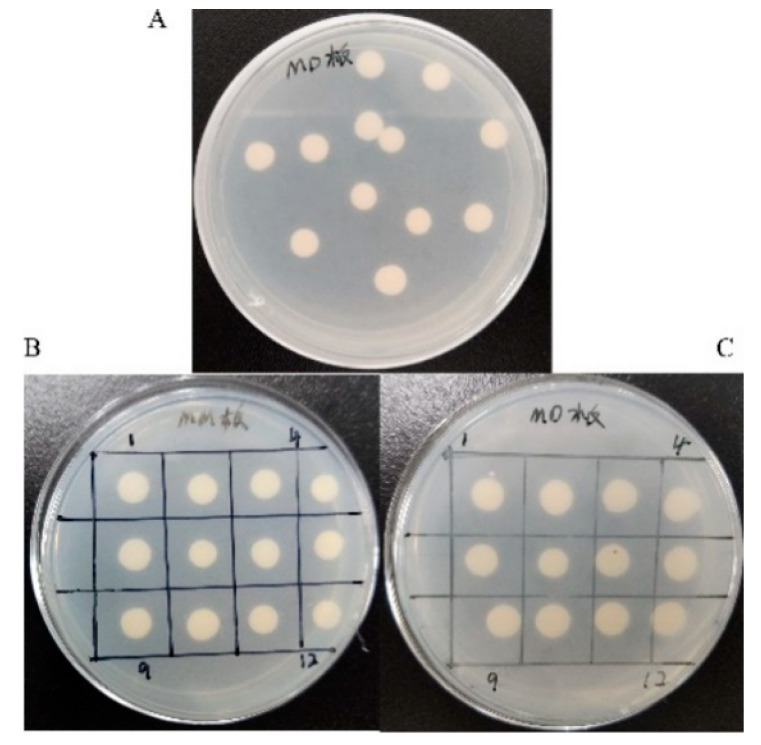
Phenotype screening of recombinant *P. pastoris* GS115. (**A**) Screening with MD media; (**B**,**C**) recombinant *P. pastoris* screening with *His^+^Mut^+^* phenotype using MM (B) and MD (C) plates.

The *P. pastoris* transformants were cultured on the plates with MD media after transformation. The transformants were transferred to the plates equipped with MM media (Figure 4). The transformants were identified with the designed primers for TV-AFB_1_D amplification. *P. pastoris* GS115 transformants with *His^+^Mut^+^* type for methanol utilization were integrated by pPIC9K-His-AFB_1_D according to the growth on the plates containing MD and MM media.

### 2.4. Recombinant TV-AFB_1_D Expression in P. pastoris GS115

**Figure 5:**
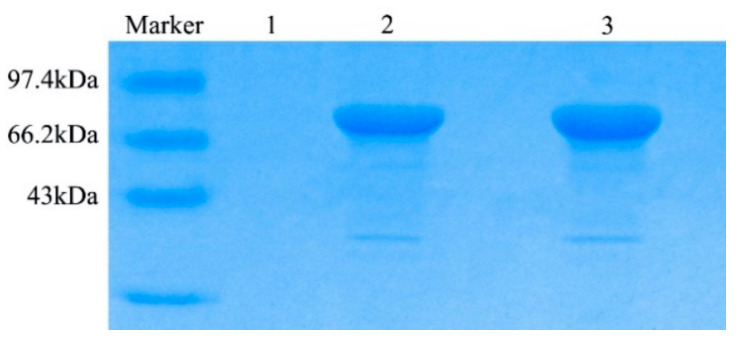
SDS-PAGE analysis of recombinant TV-AFB_1_D. Lane 1: wild-type *P. pastoris* expression; lanes 2 and 3: expression of the recombinant TV-AFB_1_D.

**Figure 6:**
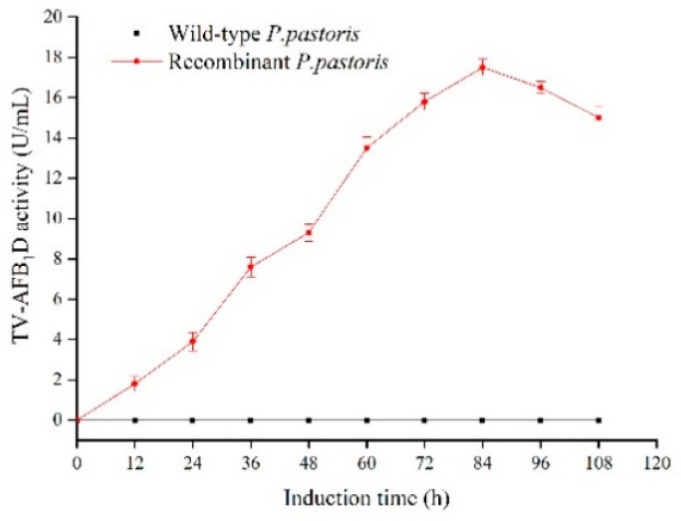
Expression of TV-AFB_1_D from the transformants and wild-type *P. pastoris.*

The recombinant TV-AFB_1_D was expressed by engineering *P. pastoris* GS115 with the size of approximately 77 kDa under the induction of 0.8% methanol. No TV-AFB_1_D was detected by the wild-type *P. pastoris* GS115 according to SDS-PAGE analysis (Figure 5). The highest activity of TV-AFB_1_D was 17.5 U/mL after the induction of 0.8% methanol (*v*/*v*) for 84 h at 30 °C. No TV-AFB_1_D activity of the fermentation broth from the wild-type *P. pastoris* GS115 was detected under the same treatment conditions (Figure 6). Therefore, the recombinant *TV-AFB_1_D* could express in engineering *P. pastoris* GS115.

### 2.5. Effect of Methanol Concentration and Time on the TV-AFB_1_D Expression

**Figure 7:**
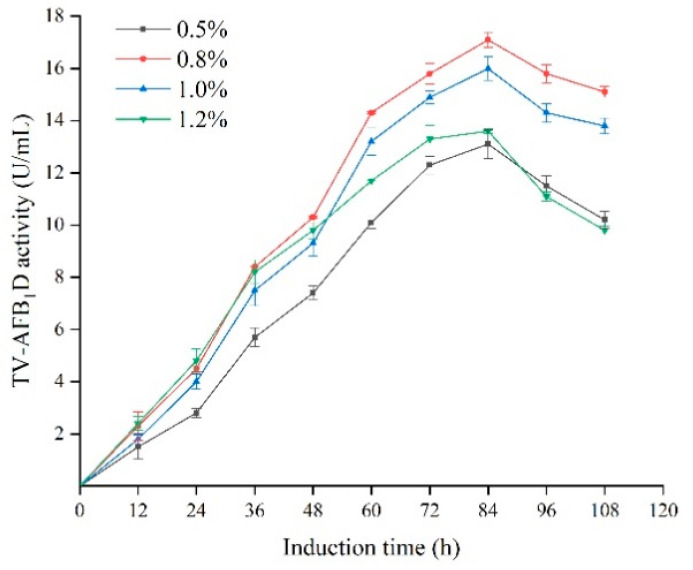
Effect of time and methanol concentration on the activity of TV-AFB_1_D.

The effects of induction time and methanol concentration on TV-AFB_1_D activity were investigated (Figure 7). The highest activities of TV-AFB_1_D expressed by the recombinant *P. pastoris* GS115 were achieved after induction for 84 h and in the presence of 0.8% methanol (*v*/*v*). In particular, the activities of TV-AFB_1_D were substantially decreased with the addition of 1.2% and 0.5% of methanol. Therefore, the optimum methanol concentration and treatment time were 0.8% and 84 h for the inducible expression of TV-AFB_1_D, respectively.

### 2.6. Effect of Temperatures and Treatment Time on the Expression of TV-AFB_1_D

**Figure 8:**
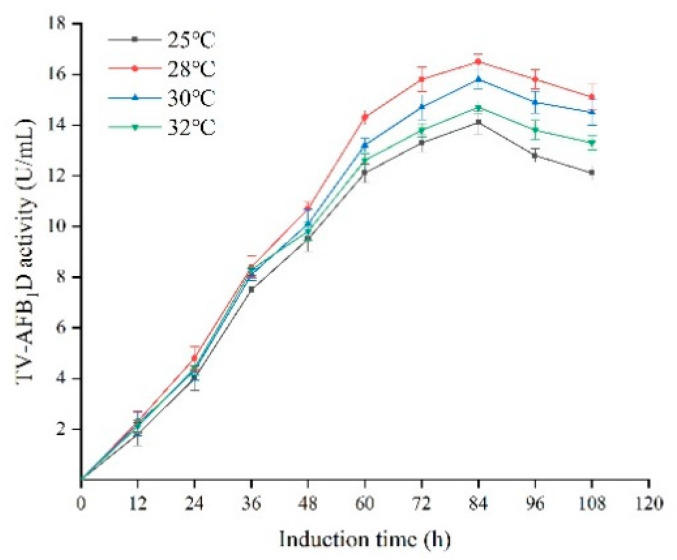
Effect of temperature on the activity of TV-AFB_1_D.

The effects of temperatures and treatment time on TV-AFB_1_D expression are shown in Figure 8. The TV-AFB_1_D activities reached the highest after induction for 84 h. The highest activity of TV-AFB_1_D was 16.5 U/mL at 28 °C among all the set temperatures of 25–32 °C. Therefore, the optimum temperature and time were 28 °C and 84 h for the inducible expression of TV-AFB_1_D in engineering *P. pastoris* GS115, respectively.

### 2.7. Product Formation of AFB_1_-Contaminated Peanuts Catalyzed by TV-AFB_1_D

**Figure 9:**
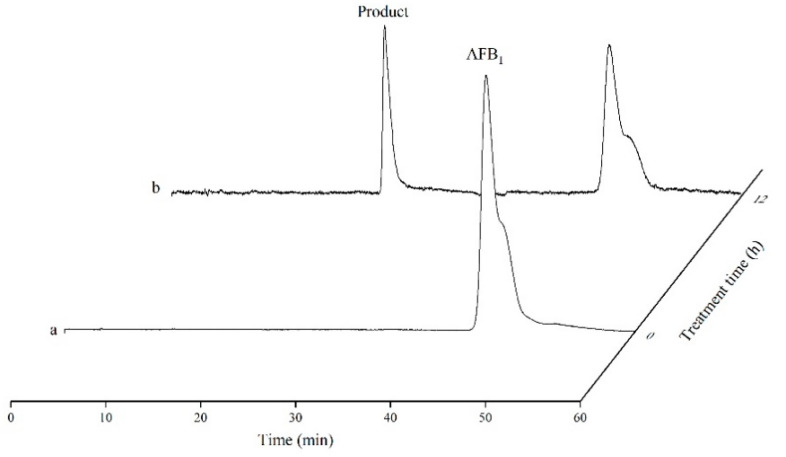
Determination of catalytic products of AFB_1_ catalyzed by TV-AFB_1_D using HPLC approach. Note: a and b represent HPLC profiles before and after catalysis, respectively.

HPLC method was used to determine the product formation of AFB_1_ catalyzed by the recombinant TV-AFB_1_D. As the control group, the AFB_1_ standard possessed a special peak after running for 45 min at a wavelength of 365 nm (Figure 9). The profiles of catalytic products were determined by HPLC after the catalysis of AFB_1_-contaminated peanuts by the recombinant TV-AFB_1_D. With the exception of the AFB_1_ standard, another peak was detected under a wavelength of 210 nm after running for 22 min. The result indicated that new product was formed under the catalysis of TV-AFB_1_D with AFB_1_ as substrate.

### 2.8. Effect of Time on the Residue of AFB_1_ Standard Sample

**Figure 10:**
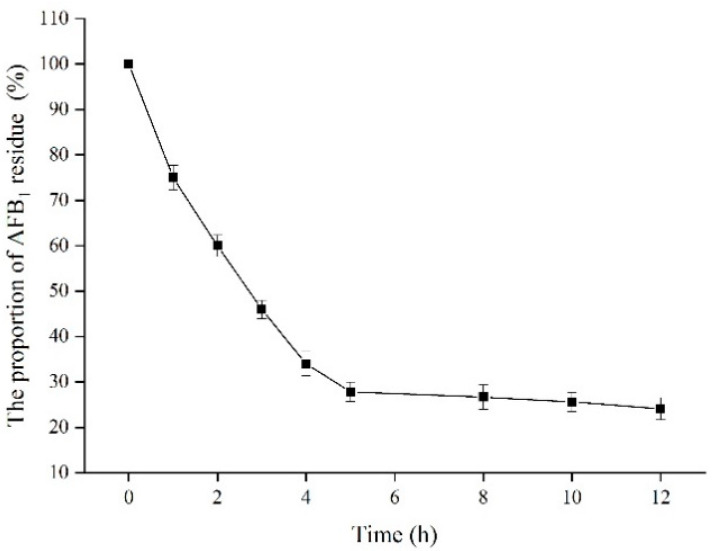
Effect of time on the concentration of AFB_1_ standard sample by TV-AFB_1_D.

The effect of catalysis time on the residual proportion of the AFB_1_ standard sample was investigated at 32 °C (Figure 10). The proportion of AFB_1_ residue substantially decreased from 100% to 27.8% for 5 h of catalysis with the degradation rate of 72.2%. During the next catalysis for 5–12 h, the proportion of residual AFB_1_ decreased slowly. The proportion of residual AFB_1_ standard sample was 24.1% after catalysis for 12 h with the degradation rate of 75.9%.

### 2.9. Effect of Time on the Proportion of AFB_1_ Residue in Peanuts

**Figure 11:**
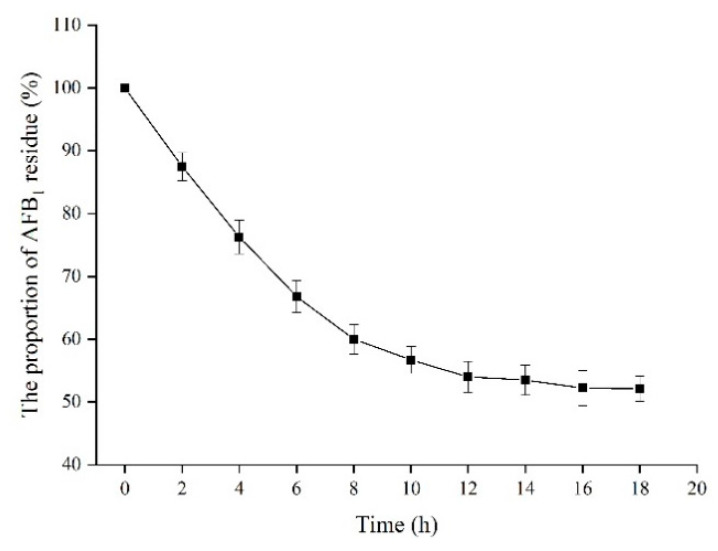
Effect of time on the concentration of AFB_1_ in AFB_1_-contaminated peanuts by TV-AFB_1_D.

The effect of treatment time on the proportion of AFB_1_ residue in AFB_1_-contaminated peanuts was investigated at 32 °C (Figure 11). Within the initial 10 h, the proportion of AFB_1_ residue decreased rapidly from 100% to 55.7%. After treatment for 18 h, the proportion of AFB_1_ residue in AFB_1_-contaminated peanuts gradually decreased to 51.5% with the degradation rate of 48.5%. Therefore, the recombinant TV-AFB_1_D could effectively catalyze AFB_1_ in AFB_1_-contaminated peanuts.

### 2.10. Effect of Temperature on the Proportion of AFB_1_ Residue in Peanuts

**Figure 12:**
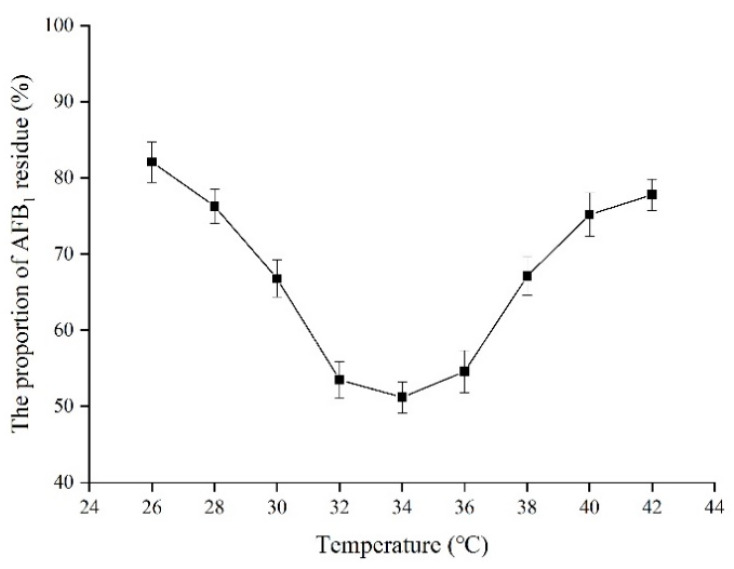
Effect of temperature on the concentration of AFB_1_ in AFB_1_-contaminated peanuts.

The effect of temperature on the proportion of AFB_1_ residue in AFB_1_-contaminated peanuts was investigated after catalysis for 5 h (Figure 12). In the range of 26 to 42 °C, the proportion curve of AFB_1_ residue was in the shape of a “V”. The proportion of AFB_1_ residue gradually decreased during the temperatures of 26 to 34 °C and increased during the temperatures of 34 to 42 °C. The lowest concentration of AFB_1_ residue in AFB_1_-contaminated peanuts was 52.1% at 34 °C with a degradation rate of 47.9%. Too low and too high temperatures were not conducive to the degradation of AFB_1_ by TV-AFB_1_D in AFB_1_-contaminated peanuts.

## 3. Discussion

Although the physical and chemical methods have been applied in the detoxification of aflatoxins, these approaches are difficult to meet the requirement of clean, safe, and environmentally friendly production [26]. Currently, the enzymatic degradation has been applied in the degradation of aflatoxins for its safety and specificity [27]. A large number of studies have been reported on the enzymes related to aflatoxin degradation (Table 1). Enzymes related to aflatoxin degradation include various catalytic properties of F_420_H_2_-dependent reductase [25], laccase [23], and peroxidase [24]. In addition, aflatoxin-detoxifizyme (ADTZ) [28], AFB_1_-horse radish peroxidase (HRP) [29], *Myxobacteria* aflatoxin degradation enzyme (MADE) [30], *Bacillus* aflatoxin-degrading enzyme (BADE) [31], and *Pantoea* aflatoxin degradation enzyme (PADE) [32] also possess the capability of aflatoxin degradation. In the previous report, the catalytic performances of enzymes were identified by using AFB_1_ standard sample as catalytic substrate. These enzymes were rarely used for degradation of aflatoxins in food and feed. In this study, *T. versicolor* aflatoxin B_1_-degrading enzyme gene was expressed in engineering *P. pastoris*. AFB_1_ standard sample and AFB_1_-contaminated peanuts were used to detect the degradation effect of AFB_1_. The AFB_1_ proportions of 75.9% and 48.5% were degraded using AFB_1_ standard sample and AFB_1_-contaminated peanuts as substrates, respectively.

## 4. Conclusions

In this study, the recombinant TV-AFB_1_D with a size of 77 kDa was effectively expressed by engineering *P. pastoris*. The optimal temperature and methanol concentration were 28 °C and 0.8% for the inducible expression of TV-AFB_1_D, respectively. The proportion of residual AFB_1_ standard sample was 24.1% after catalysis for 12 h with a degradation rate of 75.9%. In addition, the proportion of AFB_1_ residue in AFB_1_-contaminated peanuts decreased to 51.5% with a degradation rate of 48.5% after treatment for 18 h at 34 °C. This study provided an alternative TV-AFB_1_D degradation enzyme for the removal of AFB_1_ in food products and agricultural raw materials.

## 5. Materials and Methods

### 5.1. Materials and Reagents

Vector pPIC9K-His, TV-AFB_1_D gene, *P. pastoris* GS115, and *T. versicolor* were stored in the Experimental Center of Hefei University of Technology. Gene sequencing and primer synthesis were performed by Sangon Biotech. Taq polymerase, *Stu* I, *Not* I, and *SnaB* I were from NEB Company. Gel imaging systems, sodium dodecyl sulphate–polyacrylamide gel electrophoresis (SDS-PAGE), polymerase chain reaction (PCR) amplification, and electrophoresis devices were manufactured by Bio-RAD Company. ProteinIsoNi-IDA resin and AFB_1_ immunoaffinity column were from Transgen Biotech and Welch Company, respectively.

### 5.2. Construction of pPIC9K-His-TV-AFB_1_D Vector

*TV-AFB_1_D* encoding aflatoxin degradation enzyme was cloned by reverse transcription PCR (RT-PCR) technique according to Genbank accession number txid717944 in the National Center for Biotechnology Information (NCBI). The sequences of restriction endonuclease *SnaB* I and *Not* I were added in the corresponding primers for the amplification of *TV-AFB_1_D*. The corresponding primers were designed as follows: F: 5′-GGGAAA*TACGTA*ATGGCTCGCGCGAAGTACTC-3′ (*SnaB* I); R: 5′-GGGAAA*GCGGCCGC*TTAAAGCTTCCGCTCTATGA3′ (*Not* I). Both the product of amplification and pPIC9K-His vector were double digested by *SnaB* I and *Not* I. The recovered gene was ligated by T_4_ ligase to produce vector pPIC9K-His-AFB_1_D. pPIC9K-His-AFB_1_D was confirmed by PCR amplification, double-enzyme digestion identification, and gene sequencing.

### 5.3. Transformation of pPIC9K-His-TV-AFB_1_D into P. pastoris GS115

The linearized pPIC9K-His-AFB_1_D was digested by *Stu* I and integrated into *P. pastoris* GS115 by electroporation transformation method [37]. *P. pastoris* GS115 had a mutation in histidine dehydrogenase (*his4*) and could not synthesize histidine. pPIC-9K-His contained *his4* gene that was complementary to the host. The transformants were screened on the plate equipped with histidine-free medium. The recombinant *P. pastoris* with *His^+^Mut^+^* phenotype grew on minimal methanol medium (MM) plates in the presence of methanol. The transformants were screened out using the solid plates containing minimal dextrose medium (MD) and MM. The true transformants were further confirmed by PCR identification and gene sequencing.

### 5.4. Inducible Expression and Condition Optimizations of TV-AFB_1_D

The *His^+^Mut^+^* phenotype-positive clones were incubated with the shaking speed of 200 rpm at 30 °C. The collected cells of recombinant *P. pastoris* were mixed with methanol-complex buffer medium. The transformants were incubated at 30 °C with the shaking speed of 200 rpm. The fermentation broth was added with 0.8% methanol (*v*/*v*) every 24 h for the induction of recombinant TV-AFB_1_D. The parameters of temperatures, methanol concentrations, and time were optimized to enhance the inducible expression of TV-AFB_1_D.

### 5.5. Purification of Recombinant TV-AFB_1_D and Definition of Activity

The recombinant TV-AFB_1_D was purified by pre-equilibration with Ni^2+^-chelate affinity chromatography column and elution with imidazole eluent with ProteinIso Ni-IDA resin. The profile of TV-AFB_1_D protein was analyzed by SDS-PAGE. The unit of TV-AFB_1_D activity was defined as the amount of enzyme that catalyzed the consumption of 1 nmoL AFB_1_ per minute. The activity of TV-AFB_1_D was calculated by using the formula of (M_0_ − M_1_)/(312 × t × V), where M_0_, M_1_, t, and V represent the AFB_1_ weight in the control and experimental groups (µg), reaction time (min), and enzyme amount (mL), respectively; 312 indicates the molecular weight of AFB_1_.

### 5.6. Measurement of AFB_1_ Content and Degradation Products

The content of AFB_1_ was detected by HPLC using the following process parameters: methanol-to-water ratio of 40/60, isocratic elution, ultraviolet (UV) detector, C_18_ Shim-pack VP-ODS (5 µm, 250 mm × 4.6 mm), C_18_ reverse column, 0.7 mL/min of flow rate, 190–400 nm of detection wavelength, and 30 °C of column temperature. The signal of AFB_1_ was detected by the UV detector at a wavelength of 365 nm. The amount of AFB_1_ was calculated on the basis of the peak areas with the AFB_1_ standard sample as the control.

### 5.7. Application in the AFB_1_ Degradation of AFB_1_-Contaminated Peanuts

The AFB_1_-contaminated peanuts were immersed in the solution containing the recombinant TV-AFB_1_D from engineering *P. pastoris* GS115. After incubation for 12 h, the recovered peanuts were soaked with methanol solution containing 40% of water (*v*/*v*). Then, the supernatant was purified through the AFB_1_ immunoaffinity column with the flow rate of 2 drops per second. The collected eluate was further purified by the filtration with 0.22 μm size of filter membrane. The effects of treatment time and temperature on the degradation efficiency of AFB_1_-contaminated peanuts by TV-AFB_1_D were investigated.

### 5.8. Data Analysis

The data were statistically given with three replicates. Origin and SPSS software were used to analyze the data with mean ± standard deviation.

## Figures and Tables

**Table 1:** Aflatoxin-degrading enzymes and catalysis performances.

Microbial Sources and Enzymes	Conditions	Catalytic Efficiency and Time
*Mycobacterium smegmatis*, F_420_H_2_-dependent reductase [25]	20 mM Tris-HCl, pH 7.5	63%, 4 h
*Myxococcus fulvus*, MADE [30]	pH 6	96%
*Armillariella tabescens*, ADTZ [28]	0.02 M PBS, pH 6	80%
*Phanerochaete sordida*, peroxidase [33]	50 mM PBS, pH 6	86%, 48 h
*Pleurotus ostreatus*, peroxidase [34]	pH 4.5	90%, 48 h
*Bacillus subtilis*, *BsCotA* laccase [35]	50 mM Tris–HCl, pH 7.0	98%, 10 h
*Pleurotus pulmonarius*, laccase [36]	1 mM sodium acetate, pH 5	90%, 72 h
*Aspergillus flavus*, HRP [29]	1mM sodium acetate, pH 5	42%, 1 h
*Bacillus shackletonii*, BADE [31]	50 mM PBS, pH 7.0	47.5%, 72 h
*Pseudomonas aeruginosa*, PADE [32]	0.02 M PBS, pH 7	72.5%, 12h
*T. versicolor*, TV-AFB_1_D, in this study	AFB_1_ standard sample	75.9%, 12 h
*T. versicolor*, TV-AFB_1_D, in this study	AFB_1_-contaminated peanuts	48.5%, 18 h

## Data Availability

The data in this study are available in the manuscript.
